# Long‐term hepatic safety of lomitapide in homozygous familial hypercholesterolaemia

**DOI:** 10.1111/liv.15497

**Published:** 2022-12-30

**Authors:** Dominique Larrey, Laura D'Erasmo, Sallyann O'Brien, Marcello Arca, the Italian Working Group on Lomitapide, Angelo Baldassare Cefalù, Alessia Di Costanzo, Simone Bini, Antonina Giammanco, Maurizio Averna, Gabriella Iannuzzo, Giuliana Fortunato, Marco Gentile, Maria Donata Di Taranto, Arturo Pujia, Tiziana Montalcini, Chiara Pavanello, Laura Calabresi, Giovanni Battista Vigna, Marco Bucci, Katia Bonomo, Tiziana Sampietro, Francesco Sbrana, Patrizia Suppressa, Carlo Sabbà, Fabio Fimiani, Arturo Cesaro, Paolo Calabrò, Fulvio Ventura, Sergio D’Addato, Livia Pisciotta, Stefano Bertolini

**Affiliations:** ^1^ University Hospital of Montpellier Montpellier France; ^2^ Department of Translational and Precision Medicine ‘Sapienza’ University Rome Italy; ^3^ Amryt Pharmaceuticals DAC Dublin Ireland

**Keywords:** Lomitapide, hepatic, hepatic biomarkers, hepatic steatosis, homozygous familial hypercholesterolaemia, liver, liver fibrosis

## Abstract

**Introduction:**

Lomitapide is a microsomal triglyceride transfer protein inhibitor for patients with homozygous familial hypercholesterolaemia. Due to its mechanism of action, potential hepatic effects of lomitapide are of clinical interest. This study aimed to determine the long‐term hepatic safety of lomitapide.

**Methods:**

Data were aggregated from the pivotal phase 3 and extension phase clinical trial with lomitapide (median 5.1 years; serum total bilirubin, transaminases, cytokeratin‐18 [CK‐18] and enhanced liver fibrosis [ELF] score, fat‐soluble vitamins and essential fatty acids), 8‐year data from the Lomitapide Observational Worldwide Evaluation Registry (LOWER) and real‐world evidence from a cohort of patients treated with lomitapide in Italy (hepatic elastography, and FIB‐4 score for hepatic fibrosis).

**Results:**

In the phase 3 trial and the LOWER registry, any asymptomatic excursions in liver transaminase levels were not associated with elevations in bilirubin, and no Hy's law cases were detected in up to 8 years follow‐up. There were no clinically relevant increases among hepatic biomarkers CK‐18, CK‐18 fragments or ELF score and fat‐soluble vitamins and essential fatty acids remained above normal levels. In 34 patients treated in Italy with lomita pide for more than 9  years, elevations in hepatic fat were mild‐to‐moderate; hepatic stiffness remained normal, and the mean FIB‐4 score remained below the fibrosis threshold value of 2.67.

**Conclusions:**

These data indicate that the hepatic safety of lomitapide remains favourable with no clinically significant elevations in hepatic biomarkers and hepatic stiffness remained normal for more than 9 years follow‐up.

**Phase 3 trial:**

NCT00730236; extension phase: NCT00943306; LOWER: NCT02135705.

**Abbreviations**
ALT: alanine aminotransferase
AST: aspartate aminotransferase
BMI: body mass index
CI: confidence interval
CK‐18: cytokeratin‐18
DHA: docosahexaenoic acid
DILI: drug‐induced liver injury
DS: discriminative score
EPA: eicosapentaenoic acid
ELF: enhanced liver fibrosis score
FAS: full analysis set
FIB‐4: fibrosis‐4
HA: hyaluronic acid
HDL‐C: high‐density lipoprotein cholesterol
HoFH: homozygous familial hypercholesterolaemia
IQR: interquartile range
LA: lipoprotein apheresis
LFTs: liver function tests
LDL‐C: low‐density lipoprotein cholesterol
LOWER: Lomitapide Observational Worldwide Evaluation Registry
MACE: major adverse cardiovascular event
MTP: microsomal triglyceride transfer protein
NAFLD: non‐alcoholic fatty liver disease
NASH: non‐alcoholic steatohepatitis
P3NP: propeptide of type III procollagen
PBI: Pacific Biomarkers Inc.
PCSK9i: proprotein convertase subtilisin/kexin type 9 inhibitor
SD: standard deviation
TC: total cholesterol
TG: triglycerides
TIMP‐1: metalloproteinase 1
ULN: upper limit of normal
VLDL: very‐low‐density lipoproteins

**Key points**

Lomitapide is an effective treatment for patients with homozygous familial hypercholesterolaemia which acts independently of the LDL receptor. As an inhibitor of the microsomal triglyceride transfer protein, lomitapide has been associated with a mild‐to‐moderate accumulation of hepatic fat. However, this did not translate into clinically meaningful increases in biomarkers of hepatocellular damage and hepatic imaging for fibrosis remained normal during long‐term follow‐up.

## 1 INTRODUCTION

Lomitapide is a microsomal triglyceride transfer protein (MTP) inhibitor indicated as an adjunctive therapy for low‐density lipoprotein cholesterol (LDL‐C) lowering in patients with homozygous familial hypercholesterolaemia (HoFH). Lomitapide reduces LDL‐C by a mean of 50.0%–76.6%^1^, ^2^ by reducing the formation of very‐low‐density lipoproteins (VLDL) in the liver. Lomitapide has the potential to lead to idiosyncratic hepatotoxicity, and to increase hepatic steatosis by reducing the export of lipids—particularly triglycerides—from hepatocytes.^3^

Idiosyncratic hepatotoxicity is an unpredictable form of drug‐induced liver injury (DILI). It has varying delays to onset, occurs in the therapeutic dose range for the causative agent, and is characterised by high levels of liver transaminases and/or alkaline phosphatase and cholestasis.^4^ In clinical trials, the risk of significant DILI is usually estimated according to Hy's law, which identifies patients with hepatic transaminase elevation more than three times the upper limit of normal (ULN), accompanied by increases in bilirubin levels more than twice ULN. Biochemical abnormalities covered by Hy's law are associated with a 10% risk of liver failure.^5^ Importantly, statins, which are standard of care for LDL‐C lowering, have been associated with idiosyncratic hepatotoxicity; however, these are no longer considered to present a risk of clinical liver injury.^6^

Hepatic steatosis is among the most common liver abnormalities,^7^ present in 20%–30% of the general population.^8^, ^9^ Hepatic steatosis is an important element of the general sequelae of obesity, diabetes and metabolic syndrome.^10^ Carriers of common genetic variants that increase liver fat content (e.g. polymorphisms of *PNPLA3* encoding patatin‐like phospholipase domain containing‐protein 3, and *FNDC5* encoding irisin^11^, ^12^) have a higher risk of liver fibrosis^13^ and hepatocellular carcinoma than in those without such mutations.^14^ Carriers of nonsense mutations for *APOB* also have a higher risk of hepatocellular carcinoma.^15^ Hepatic steatosis, with or without steatohepatitis and liver injury can also be caused by drugs such as glucocorticoids, methotrexate, tamoxifen, valproic acid, amiodarone and chemotherapy.^16^ According to drug type and mechanisms of accumulation of lipids in hepatocytes, bland steatosis may evolve into non‐alcoholic fatty liver disease, inflammation and/or fibrotic cirrhosis.^17^

None of the types of long‐term liver damage outlined above have been observed during the clinical use of lomitapide in HoFH. However, as discussed, lomitapide has the potential to cause hepatic steatosis, and the question remains whether there is the development of steatohepatitis and subsequent liver fibrosis or cirrhosis over the long term. The current report includes previously unpublished hepatic safety data from several sources with the aim of gaining a better understanding of the long‐term hepatic safety of lomitapide.

## 2 PATIENTS AND METHODS

The data described in this paper are derived from three different sources (Table S1).

### 2.1 Phase 3 clinical trial and long‐term extension study of lomitapide in patients with HoFH

The details of phase 3 clinical trial and its long‐term extension have been published before.^1^, ^18^ In brief, 29 adult patients with HoFH were commenced on lomitapide 5 mg/day in the 78‐week phase 3 study. Doses were escalated to a maximum‐tolerated dose. Patients continued standard lipid‐lowering therapy, including lipoprotein apheresis (LA). In accordance with the approved product label for lomitapide, all patients received essential fatty acid and vitamin E supplements and were instructed to follow a diet in which <20% of energy was derived from fat. Nineteen of the 23 patients who completed the phase 3 trial entered the long‐term extension trial. Overall, the exposure to lomitapide across both trials was median of 5.1 years. Blood samples were collected for laboratory analysis according to the study visit schedules as described previously.^1^, ^18^

Data from the clinical trial programme were used to evaluate mean liver transaminase levels and instances of Hy's law. For biomarker analysis, stored serum samples from the phase 3 trial were analysed by Pacific Biomarkers Inc (PBI). Measurements were conducted on cytokeratin‐18 (CK‐18), CK‐18 fragments, tissue inhibitor of metalloproteinase 1 (TIMP‐1), amino‐terminal propeptide of type III procollagen (P3NP) and hyaluronic acid (HA). Upper and lower limits of quantitation for the assays were determined by PBI. For CK‐18 and CK‐18 fragments, duplicate tests were conducted on matching aliquots 6 months apart to assess the stability of the samples in cold storage.

Values for TIMP‐1, P3NP and HA can be used to calculate an individual patient's enhanced liver fibrosis (ELF) score. The ELF score correlates with the degree of fibrosis in chronic liver disease.^19^

In the present study, ELF was calculated using the formula provided by Guha et al^20^ as:

$$\begin{array}{c} \mathrm{ELF}=–7.412+\left(\ln\left(\mathrm{HA}\right)\times 0.681\right)+\left(\ln\left(P3\mathrm{NP}\right)\times 0.775\right)\\ \;+\left(\ln\left(\text{TIMP}1\right)\times 0.494\right). \end{array}$$

ELF scores derived by this method have a negative value. For NAFLD, indication of some degree of fibrosis is suggested by values greater than −0.6415. For ELF scores, above this figure, there is a 72% positive and 66% negative predictive value for fibrosis with a sensitivity of 80% and a specificity of 56%.^20^

Values for HA, P3NP, TIMP‐1 and ELF during lomitapide treatment were compared to those at baseline using a paired t‐test.

Fat‐soluble vitamins and essential fatty acids were also measured as patients receiving lomitapide need to be on a low‐fat diet with <20% energy from fat due to the inhibitory effect of lomitapide on chylomicron formation in the intestine. It is important that these micronutrients are maintained at or above the normal level. Vitamins A, vitamin D and beta‐carotene were assessed by measuring serum concentrations of the individual nutrients. Vitamin E was reported as the ratio of vitamin E/total lipids (total cholesterol plus triglycerides). Levels of vitamin K were assessed directly by measuring the ratio of uncarboxylated osteocalcin/total osteocalcin. The fatty acid profile included serum levels of all essential fatty acids. All patients receiving lomitapide also received vitamin E and essential fatty acid supplements (daily amounts: vitamin E 400 IU, eicosapentaenoic acid [EPA] 110 mg, docosahexaenoic acid [DHA] 80 mg, alpha‐linolenic acid 220 mg and linoleic acid 200 mg).

### 2.2 Lomitapide Observational Worldwide Evaluation Registry (LOWER)

LOWER is a global, prospective, observational registry assessing long‐term safety and effectiveness of lomitapide in clinical practice.^21^ LOWER has been designed to enrol 300 adult HoFH patients treated with lomitapide and followed for a minimum of 10 years. The present analysis from LOWER includes 214 HoFH patients in the full analysis set (FAS), representing a maximum of 8 years' exposure to lomitapide (mean 34.5 months). Available data from LOWER are included to provide additional, long‐term assessment of liver transaminases and instances of Hy's law cases. Liver transaminase data were collected during routine laboratory assessments and analysed in the same manner as for the phase 3 study.

### 2.3 Italian patient cohort sub‐analysis from the Pan European Observational Study conducted by the Italian and European Working Group on Lomitapide in HoFH

The Pan European Observational Study has been published previously.^22^ Seventy‐five HoFH patients in Europe receiving lomitapide according to the product label in a real‐world clinical setting were evaluated for long‐term effectiveness and safety of lomitapide. The sub‐set of 34 HoFH patients enrolled in Italy were included in the present analysis. For descriptive statistics, continuous traits were presented as mean and standard deviation or as median and interquartile range (IQR) as appropriate.

Investigators were asked to retrieve all available hepatic ultrasound and elastography performed on the dates closest to the baseline and last follow‐up visit. The degree of hepatic steatosis was classified as mild, moderate or severe according to the criteria of local examiners. Liver elastography was measured and reported as velocity of transmission of a shear wave through the liver in kPa. Normal elastography results are in the range of 2–7 kPa.^23^

Laboratory tests were conducted according to each patient's individual visit schedule. Serum or plasma samples were analysed using local chemical pathology laboratory protocols. Among the laboratory parameters collected, hepatic biomarkers were analysed, including Fibrosis‐4 (FIB‐4). The FIB‐4 score is a simple, non‐invasive biomarker for liver fibrosis.^24^ The test produces an overall score based on the following formula: age (years) × aspartate aminotransferase (AST) [IU/L]/(platelets [10^9^/L] × (alanine aminotransferase [ALT] [IU/L])^0.5^).^24^ The FIB‐4 index has an area under the receiver operating characteristic curve of 0.76. A threshold value of <1.30 has a negative predictive value of 90% for the exclusion of extensive fibrosis (F4‐F6 in the Ishak classification). A threshold value of >2.67 has a positive predictive value of 80% for the diagnosis of extensive fibrosis.^24^, ^25^ FIB‐4 scores were compared between baseline and follow‐up using a paired t‐test.

Regardless of study, all patients provided an informed consent. All studies were conducted according to the principles of the Declaration of Helsinki.

## 3 RESULTS

### 3.1 Transaminases and Hy's law analysis

**FIGURE 1:**
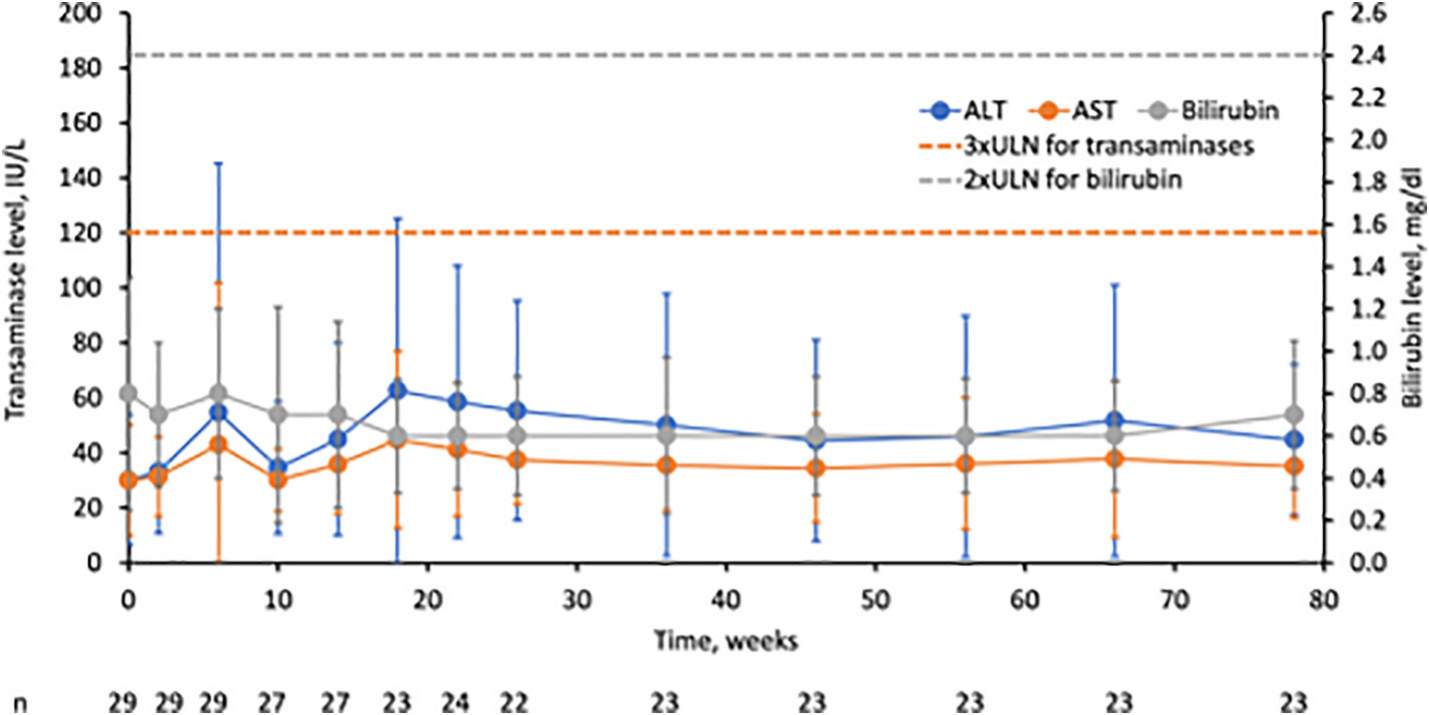
Mean aminotransferase and bilirubin levels from the pivotal phase 3 trial in HoFH. Values represent means ± standard deviation. ULN for bilirubin (all patients) is 1.2 mg/dl; ULN for ALT in females is 33 and males is 40 IU/L; ULN for AST in females is 36 and males is 43 IU/L. ALT, alanine aminotransferase; AST, aspartate aminotransferase; ULN, upper limit of normal.

Twenty‐nine HoFH patients in the pivotal phase 3 trial (follow‐up ≤1.5 years) were monitored for elevations in transaminases and any possible elevations in bilirubin (Figure 1). Mean increases from baseline to weeks 26 and 78 were noted for both ALT and AST; the mean increases for ALT (19.3 and 15.0 U/L to weeks 26 and 78, respectively) were greater than those observed for AST (6.8 and 8.9 U/L, respectively). Bilirubin levels were <2× upper limit of normal value (ULN) at all time points for patients with ALT and/or AST elevations ≥3 × ULN; therefore, no patients met the criteria for Hy's law during the clinical trial development programme (Table S2).

In addition, a modest and gradual decrease in mean body weight (from 73.5 ± 18.1 kg at baseline to 67.8 ± 13.0 at week 78; p = .004) was observed (Figure S1). Results for body mass index (BMI) mirrored those for weight. At weeks 26 and 78, mean BMI had reduced by −1.2 kg/m^2^ (−4.7%) and − 0.9 kg/m^2^ (−3.1%) from baseline. No patients were underweight (BMI <18.5 kg) at study entry. Importantly, none of the patients had BMI <18.5 kg/m^2^ at any time during the study, indicating that observed weight loss was not of clinical concern for any patient. Moreover, for patients who were overweight or obese at baseline, weight losses were in the range indicative of clinical benefit.

Similarly, among the 214 patients in the FAS of the LOWER registry treated over an 8‐year period with lomitapide 5–40 mg/day (Figure S2; Table S3), no Hy's law cases were reported since the registry began in 2014. The number of events in LOWER at each timepoint shows that the majority of first events occurred within the first 12 months of treatment which coincides with the period of lomitapide dose titration. Cumulatively, 42 of 214 patients (19.6%) experienced elevated ALT or AST (≥3× ULN), while the majority of patients did not have elevated transaminases after initiation of lomitapide treatment. All first events of elevated liver function tests (LFTs) occurred within 20 months after the start of lomitapide treatment (Figure S2). The number of hepatic transaminase elevations >3x ULN in LOWER that persisted >4 weeks despite dose reduction or interruption has remained unchanged for 3 years, occurring in 19 patients (8.9%).

In the 34 Italian patients reported here (genetic status reported in Table S4), over a maximum of 9.5 years follow‐up (mean ± SD 34.0 ± 36.3 months, median 32.1 months), mean on‐treatment AST levels were 38.8 ± 19.0 IU/L (range 16.2–89.0 IU/L; interquartile range [IQR] 21.6–51.1 IU/L), and corresponding ALT levels were 48.5 ± 40.2 IU/L (11.3–229.0 IU/L; IQR 24.4–62.6 IU/L). Among these patients, one experienced an increase in liver function test >10xULN without any increase in serum bilirubin. This patient temporarily stopped all treatment and subsequently resumed treatment with lomitapide without experiencing any further increases in liver function tests. Another patient showed an elevation between 5x and 10x ULN after 15 months without an increase in serum bilirubin. The patient did not undergo any changes in therapy. After this episode, the same patient experienced fluctuations in LFTs between normal values and <10x ULN. At the last follow‐up, LFTs were normal. A further patient experienced an elevation of LFTs between 3x and 5x ULN after 15 months of treatment, which returned to normal range at the 18‐month visit (no information on serum bilirubin)—but the patient stopped lomitapide at that point.

### 3.2 Biomarkers of hepatic safety

**FIGURE 2:**
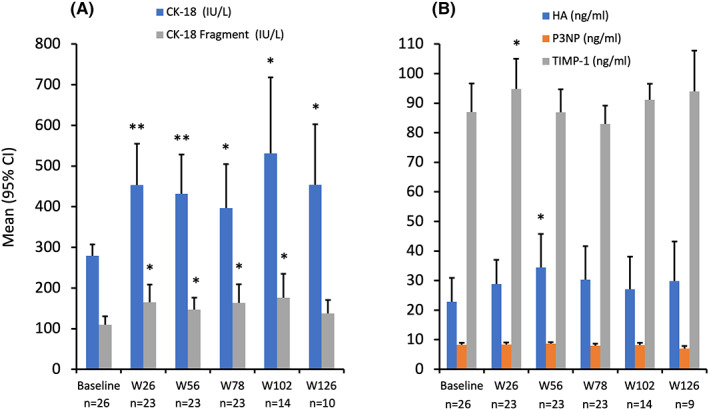
CK‐18 fragments (A) and ELF component (B) biomarkers during lomitapide treatment in the long‐term extension study. Values represent mean (95% CI) CK‐18 and extracellular matrix markers in patients with follow‐up data. *p < .05; **p < .01. CK‐18, cytokeratin‐18 fragment; ELF, enhanced liver fibrosis; HA, hyaluronic acid; P3NP, amino‐terminal propeptide of type III procollagen; TIMP‐1, tissue inhibitor of matrix metalloproteinase; W, week.

During treatment with lomitapide in the pivotal phase 3 and extension trials, circulating levels of CK‐18 and CK‐18 fragments were measured as biomarkers of hepatic inflammation and indicators of non‐alcoholic steatohepatitis (NASH). Mean levels of CK‐18 and CK‐18 fragments exhibited statistically significant mean increases from baseline to week 26, and then remained relatively stable between weeks 26 and 126 (Figure 2A). At no time did the mean levels of CK‐18 fragments exceed the suggested thresholds for NAFLD and definite NASH (230 and 270 U/L, respectively).

For HA and TIMP‐1, statistically significant mean elevations over baseline were only evident for HA at week 56 (12.4 ng/ml [95% CI: 1.5, 23.3] *p* = .03) and TIMP at week 26 (mean change from baseline 12.9 ng/ml [95% CI: 0.2, 25.6]; *p* = .04). At baseline, the mean P3NP value was 8.24 ng/ml. Thereafter, mean P3NP values never exceeded 8.66 ng/ml at weeks 26, 56, 78 and 102, and were even lower at week 126 (7.01 ng/ml). There were no significant changes from baseline observed with P3NP (Figure 2B). Calculation of the mean change from baseline in ELF scores based on these markers demonstrated increases at weeks 26 (0.32 [95% CI: 0.06, 0.60]; *p* = .02) and 56 (0.41 [95% CI: 0.18, 0.64]; *p* = .001), but not at week 78 (0.20 [95% CI: −0.08, 0.49]; *p* = .15; Figure S3), indicating no progressive elevation over time.

Evaluating ELF at the individual patient level showed that there were five patients with ELF scores >−0.6415 post‐baseline through week 78. These elevated scores were mostly at single timepoints—one patient at week 26 (−0.5510), two patients at week 56 (−0.3357 and −0.5731), one patient at week 78 (−0.4715), and one patient at weeks 26, 56 and 78 (−0.3317, −0.3787 and −0.1195). For this latter patient, hepatic fat content was 0.86% at baseline, 11.85% at week 26 and 0.39% at week 56, indicating that NAFLD was not present in this patient. One subject had ELF >−0.6415 at baseline but not thereafter. No subjects exhibited ELF scores that would exceed the threshold whereby the odds of having either moderate or severe fibrosis would exceed the odds of not having moderate or severe fibrosis. During the study, none of the patients underwent post‐baseline liver biopsy due to liver‐related safety concerns.

### 3.3 FIB‐4 scores

**FIGURE 3:**
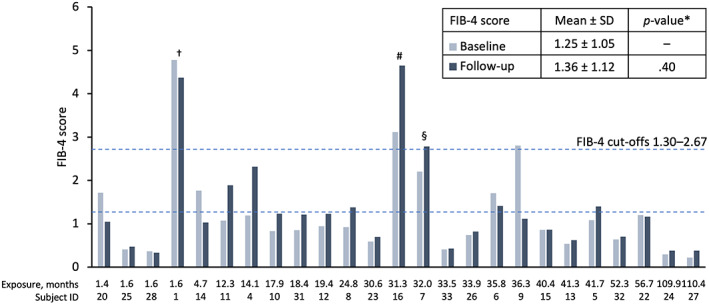
FIB‐4 scores by months of treatment with lomitapide in patients with baseline and follow‐up data (*n* = 25). Bars represent individual patient data at baseline and last follow‐up. FIB‐4 score is calculated as age (years) × AST [IU/L]/(platelets [10^9^/L] × (ALT [IU/L])^0.5^).^24^ *Parametric, two‐tailed t‐test. SD, standard deviation; ^†^77‐year‐old patient with short follow‐up; ^#^patient with very low platelet counts at follow‐up (74 × 10^9^/L), ^§^66‐year‐old patient.

In the present study, FIB‐4 scores were calculable in 25 patients belonging to the Italian cohort with baseline and follow‐up data on platelet levels and transaminases, ALT and AST. FIB‐4 results showed no significant increase in mean FIB‐4 scores from baseline for up to 110.4 months (>9 years; exposure range 1.4–110.4 months, median exposure 31.3 months; Figure 3; *p* = .40). FIB‐4 scores remained below the 2.67 level predictive for significant fibrosis in most patients. Additionally, 72% and 68% of patients had FIB‐4 scores <1.3 at baseline (*n* = 18) and follow‐up (*n* = 17), respectively, whereby scores below this level are 90% predictive of no fibrosis in patients aged 35–65 years old.^25^, ^26^

One patient (patient 1) had elevated baseline and follow‐up FIB‐4 scores >2.67 (4.78 and 4.37, respectively). A further patient (patient 7) with an elevated baseline FIB‐4 score of 2.20 showed a modest increase to >2.67 (2.79) at follow‐up. In these two cases, the patients were > 65 years old (77 and 66 years old, respectively; Figure 3; patients 1 and 7).

Patient 16 also had elevated baseline and follow‐up FIB‐4 scores >2.67 (3.12 and 4.65, respectively; Figure 3). This patient was 41 years old, but had low platelet counts at baseline and last follow‐up, resulting in an elevated FIB‐4 score.

In all cases, elevated FIB‐4 scores were set against a background of normal liver transaminases. For patients 7 and 16, FIB scores >2.67 were associated with normal hepatic stiffness measured by elastography <7.0 kPa (Table S6). Hepatic stiffness data were not available for patient 1. Note that 10 of the 25 patients with FIB‐4 data (40%) had less than 24 months of follow‐up.

### 3.4 Hepatic Imaging

The baseline characteristics of 34 HoFH patients from the Italian cohort are reported in Table 1. The median duration of treatment was 2.4 ± 2.6 years (IQR 0.7–3.2) with a maximum follow‐up of 9.8 years. Mean on‐treatment lomitapide dose by patient was 15.6 ± 12.5 mg/day (min 4.8; max 55.2).

**TABLE 1:** Baseline characteristics of a real‐world cohort of HoFH patients from Italy

Lomitapide cohort (*N* = 34)		
Demographic		
Age, years (IQR)	40.0 (26.0–52.7)	
Male, *n* (%)	18 (52.9)	
Risk factors		
BMI, kg/m^2^ (IQR)	23.7 (21.1–26.8)	
MACE, *n* (%)	19 (55.9)	
Baseline lipid profile	mg/dl	mmol/dl
TC, median (IQR)	349.5 (255.5–476.5)	9.0 (6.6–12.3)
LDL‐C, median (IQR)	276.5 (196.0–386.2)	7.2 (5.1–10.0)
HDL‐C, median (IQR)	45.0 (34.7–51.2)	1.2 (0.9–1.3)
TG, median (IQR)	96.5 (67.7–132.0)	1.1 (0.8–1.4)
Lipid‐lowering therapies		
Statin, *n* (%)	32 (94.1)	
Ezetimibe, *n* (%)	29 (85.3)	
Apheresis, *n* (%)	12 (35.3)	
PCKS9i, *n* (%)	6 (17.6)	

Abbreviations: BMI, body mass index; HDL‐C, high‐density lipoprotein cholesterol; IQR, interquartile range; LDL‐C, low‐density lipoprotein cholesterol; MACE, major adverse cardiovascular event; PCSK9i, proprotein convertase subtilisin/kexin type 9 inhibitor; TC, total cholesterol; TG, triglycerides.

Ultrasound and transient elastography were used to evaluate hepatic steatosis and hepatic stiffness (fibrosis), respectively. Of the total cohort (*n* = 34), 28 patients had follow‐up hepatic ultrasound measurements, and 22 patients had measurements at both baseline and follow‐up. From the cohort of 28 patients 32.1% had mild hepatic fat (*n* = 9) and 39.3% had moderate hepatic fat (*n* = 11) at follow‐up. Of the 22 patients with both measurements available, 68.2% patients (*n* = 15) experienced no change in hepatic steatosis (Table 2). Among the seven patients (31.8%) who had increases in steatosis, there was no evident association with the duration of lomitapide exposure or lomitapide dose (Table S5; Figure S4).

**TABLE 2:** Change in ultrasound data in the Italian cohort (*n* = 22)

Change	Patients, % (*n*)
Increase	31.8 (7)
No change	68.2 (15)
Decrease	0.0 (0)

*Note*: A graphical representation of these data by patient is given in Figure S4.

**FIGURE 4:**
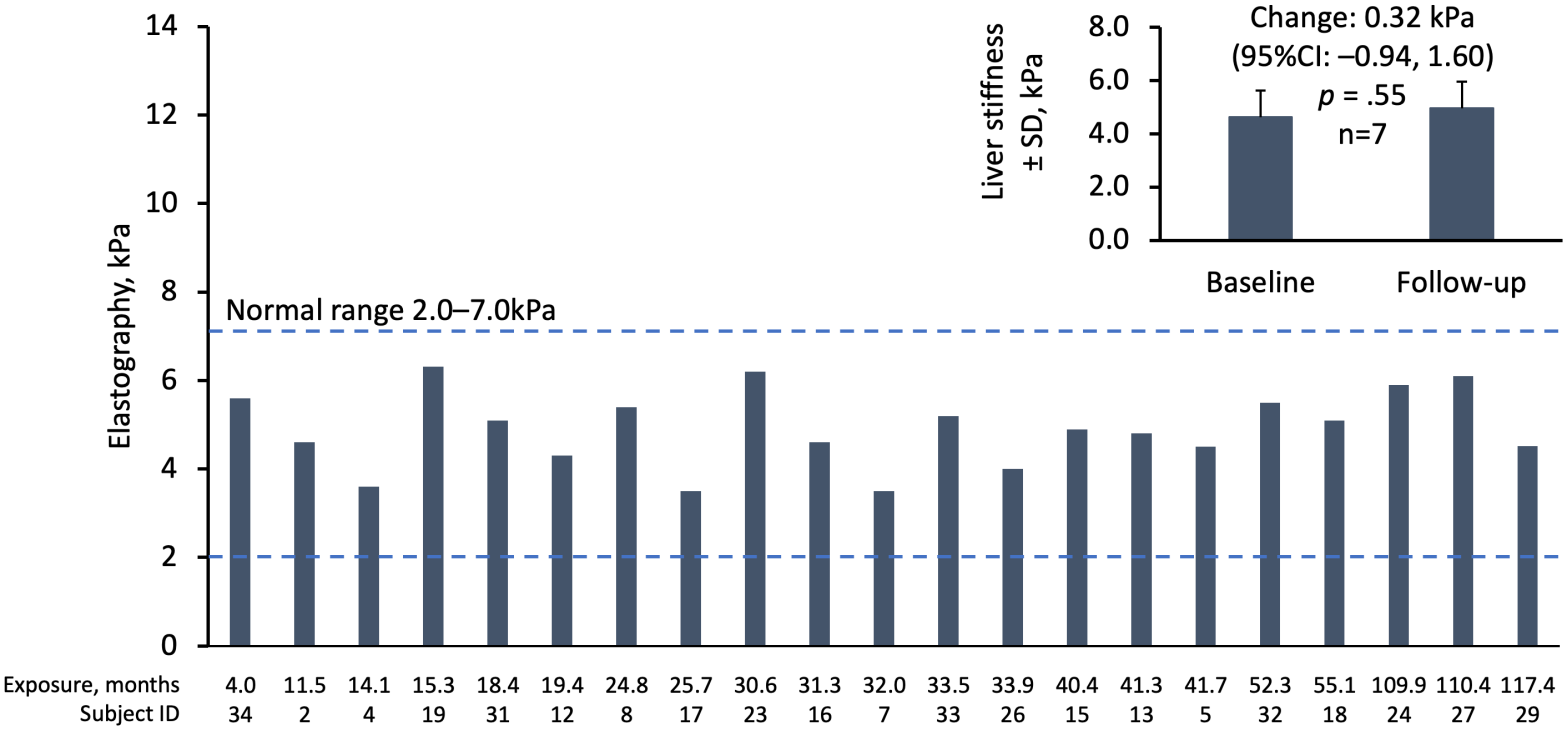
Hepatic stiffness according to liver elastography by months of exposure to lomitapide in patients with follow‐up elastography data (*n* = 21). Bars represent individual patient data at last follow‐up visit. Inset: mean ± SD liver stiffness at baseline and follow‐up for *n* = 7 patients with baseline and follow‐up data. SD, standard deviation.

In patients with elastography data at last follow‐up visit (n = 21), hepatic stiffness was normal in all patients treated with lomitapide for up to 117.4 months (9.8 years) with a mean of 4.9 ± 0.9 kPa (Figure 4). Baseline data (before lomitapide) and follow‐up data were available in seven patients in whom the mean change in liver stiffness was 0.32 kPa (95% CI −0.94, 1.60). This difference was not statistically or clinically significant (*p* = .40; Figure 4 inset). No patients had hepatic stiffness measurements above the threshold of 7.0 kPa at last follow‐up (Figure 4), and all patients with elevated FIB‐4 scores had normal hepatic stiffness as measured by elastography (Table S6).

### 3.5 Fat‐soluble vitamins and essential fatty acids

In the phase 3 trial, levels of fat‐soluble vitamins remained relatively stable through week 78 of phase 3 study. Mean vitamin E levels (which were supplemented with 400 IU vitamin E daily) decreased by −1.2 mg/dl from baseline to weeks 26 and 78, but values did not fall below the normal range (Table S7). None of the subjects had a vitamin E/total lipid ratio <1 at any time.

The fat‐soluble vitamins A, D and K, and beta‐carotene, which were not supplemented in the diet, were measured throughout the study. Mean levels of vitamins A, D and K, increased from baseline to weeks 28 and 78, while beta‐carotene decreased slightly or remained unchanged. Mean levels of essential fatty acids (supplemented in the diet according to the prescribing information) decreased over time compared to baseline in the phase 3 study. However, there were no shifts to below‐normal levels in the fatty acids linoleic acid, alpha‐linolenic acid, arachidonic acid, EPA and DHA during 78 weeks of treatment (Table S8).

### 3.6 Apheresis schedules

In the phase 3 study, 18 (62.1%) patients were on LA at baseline. At week 26, three (16.7%) had stopped LA and three (16.7%) had reduced their apheresis frequency. In the Italian cohort, median LDL‐C levels decreased by 58% from 276.5 mg/dl (IQR 196.0–386.2) to 131.0 mg/dl (66.6–228.5) at last visit (*n* = 34). At 5 years mean LDL‐C levels decreased by 65.6% (IQR 59.4–83.3) in seven patients with evaluable data to 97 mg/dL (IQR 40–155). Of the 34 patients in the total cohort, 12 patients were receiving LA at baseline and eight (67%) were able to stop LA after lomitapide treatment. A further three patients (25.0%) were able to reduce LA frequency from once every 2 weeks to once every 3–4 weeks due to effective pharmacological LDL‐C lowering therapy.

## 4 DISCUSSION

In the present study, we examined the long‐term hepatic safety of lomitapide based on a range of data available from various sources, which enabled the evaluation of follow‐up for more than 9 years. The data show that lomitapide results in some hepatic alterations, but that overall, there were no clinically meaningful signals of liver damage.

Liver transaminases are routinely measured in patients receiving lomitapide for HoFH. In the present analysis of data from multiple sources, a minority of patients experienced increases in liver transaminase levels >3xULN. In LOWER, the majority of first events occurred within the first 12 months of treatment and all first events occurred within 20 months, which coincides with the period of lomitapide dose titration. The transaminase profile for lomitapide is similar to the profile seen in the early years with statins and has not been associated with an increase in other hepatic events with either medication. The number of hepatic transaminase elevations >3x ULN that persist >4 weeks despite dose reduction or interruption has remained unchanged since 2019.^27^ In the data shown here, we have included the corresponding data on circulating levels of bilirubin from the phase 3 trial and the extension phase study where there were no concurrent increases in bilirubin, and therefore no cases of Hy's law emerged in any patient receiving lomitapide. These findings were confirmed by long‐term data from LOWER with up to 8 years' follow‐up.

Evaluation of liver safety included assessment of biomarkers of liver inflammation, CK‐18 and CK‐18 fragments, in samples taken from patients in the phase 3 trial up to 126 weeks (>2 years). Studies have demonstrated that circulating levels of CK‐18 and CK‐18 fragments are correlated with the degree of hepatocyte apoptosis.^28^, ^29^, ^30^ Studies suggest that CK‐18 fragments can differentiate between NASH and NAFLD,^30^, ^31^, ^32^ with increases in hepatocellular apoptosis present in NASH, but not in NAFLD.^33^ Optimal cut‐off‐points of serum CK‐18 fragments suggested for NAFLD and definite NASH range from 230 to 270 U/L, respectively.^34^, ^35^ At no time point did the mean CK‐18 fragments levels exceed these thresholds in our analysis. Mean increases were noted in CK‐18, and CK‐18 fragments versus baseline but these increases did not continue to increase over time. We conclude that these results do not suggest progressive liver injury over the course of the study. It should be noted that suggested thresholds vary quite markedly,^36^, ^37^, ^38^, ^39^, ^40^, ^41^ and in most studies and systematic reviews, researchers suggest that CK‐18 fragment results are viewed alongside a range of other measures (e.g. liver biopsy) to determine the degree of hepatocyte inflammation.^42^

Levels of HA, P3NP, and TIMP, and the ELF score (which combines all three independent markers) have been shown to correlate with the extent of hepatic fibrosis in several different conditions.^19^, ^20^, ^43^, ^44^ ELF scores derived from the HA, P3NP and TIMP levels collected in the phase 3 study showed statistically significant increases from baseline at weeks 26 and 56, but not at week 78. This finding was underpinned by the fact that there were only significant mean elevations for HA at week 56 and for TIMP at week 26 – but no significant alterations of the markers at any other time point. Individual increases observed in the ELF score above the threshold of −0.6415 were mainly at single timepoints or at baseline only. There were no consistent patterns in ELF score elevations over time. Only one patient had an increase in ELF score at weeks 26–78. This patient did not have NAFLD at baseline and only had a temporary increase in hepatic fat at week 26 which decreased to 0.39% at week 56. As the ELF test has been validated in patients with NAFLD, its usefulness in non‐NAFLD patients may be limited. It is important to note that the high sensitivity of the test (80%) using a cut‐off of −0.6415, comes at the expense of limited specificity (56%), which means there will be a substantial number of false‐positive results. Overall, these results indicate that there was no definitive pattern to alterations in ELF or its component markers and no subject exhibited values indicative of moderate or severe fibrosis.

The biomarker results were further supported by the analysis of FIB‐4 scores. FIB‐4 score is a useful, non‐invasive marker of liver fibrosis that uses a calculation based on platelet and transaminase levels to provide a validated measure of the risk of liver fibrosis.^24^, ^25^ A FIB‐4 score <1.3 is predictive of no fibrosis in patients aged 35–65 years old and <2.0 for those aged ≥65 years.^25^, ^26^

A FIB‐4 score above 2.67 is considered predictive for clinically meaningful fibrosis in NAFLD in all age groups >35 years old.^25^, ^26^ In the analysis presented here, there was no significant change in the mean FIB‐4 score from baseline to last follow‐up. However, when analysing the individual patient FIB‐4 results, we noted three outliers. In two cases, the patients were >65 years old (77 and 66 years old, respectively; Figure 3; patients 1 and 7). The 77‐year‐old patient (patient 1) had an elevated baseline FIB‐4 score of 4.78 (pre‐lomitapide treatment) which decreased slightly post lomitapide to 4.37. The patient who was 66 years old (patient 7) had an elevated baseline FIB‐4 score of 2.2, with a modest elevation to >2.67 (2.79) at follow‐up. However, in both cases, the patients had normal transaminase values and patient 7 had normal hepatic elastography results. Elastography was not available in patient 1. The third patient, who was 41 years old (patient 16), had low platelet counts and an elevated FIB‐4 score at baseline (3.12) and at last follow‐up (4.65). The FIB‐4 score becomes elevated when very low platelet counts are among the input variables.^24^ Notably, this patient with a low platelet count had normal ALT and AST levels and a normal elastography reading at last follow‐up. In common with all the patients with elastography data at follow‐up, this reading was below the 7.0 kPa threshold level indicative of fibrosis, thereby indicating that this patient is unlikely to have hepatic fibrosis and the low platelet count may be due to an alternative aetiology. FIB‐4 scores should not be calculated using acute changes in ALT or AST, as this will result in a falsely elevated score. Rather, FIB‐4 should be calculated using stable ALT and AST values, reflecting the overall hepatic status of the patient. It is important to note that the biomarker scores have been designed for long‐term monitoring in the setting of NAFLD.^45^ The marker has not been validated in HoFH patients who may or may not develop varying levels of hepatic steatosis, nor has it been validated in situations in which pharmacological challenge can result in rapid and transient alterations to LFTs.

Data from the Italian cohort also provided the opportunity to evaluate the effect of lomitapide on the liver using hepatic imaging (ultrasound and/or elastography) in patients exposed to long‐term treatment (more than 9 years). There was evidence of a mild to moderate increase in hepatic fat, which is not unexpected due to the mode of action of lomitapide that reduces the formation of VLDL and thereby the export of triglycerides from the liver, leading to a reduction in LDL‐C. However, elastography data demonstrated that the mild to moderate increase in hepatic fat observed in the HoFH patients treated with lomitapide did not translate into an increase in hepatic stiffness or fibrosis. Therefore, the long‐term, real‐world evidence in this cohort of 34 patients with >9 years support the notion that there are no signs of steatohepatitis or fibrosis in HoFH patients treated with lomitapide to date.

The results described above indicate that, despite alterations in some liver parameters with lomitapide treatment, no HoFH patient progressed to develop NASH or cirrhosis. One hypothesis is that the mechanism of hepatic steatosis seen in some patients receiving lomitapide may be different to hepatic fat accumulation consequent to metabolic syndrome or genetic conditions. Hepatic fat accumulation is multi‐factorial, and contributing processes include MTP inhibition (e.g. lomitapide^3^), inhibition of VLDL secretion by other drugs, for example corticosteroids,^46^ or genetic variants,^47^ inhibition of beta‐oxidation of fatty acids, for example amiodarone^48^ and insulin resistance.^49^ Additionally, the inflammatory processes stimulated by hepatic steatosis may be modulated by lipotoxicity associated with abdominal fat deposits outside the liver.^50^, ^51^ This milieu of interacting processes is likely to contribute to the fact that hepatic steatosis does not necessarily result in systemic inflammation, and thereby supports the proposal that accumulation of hepatic fat in lomitapide‐treated patients may have a different metabolic and clinical course to NAFLD in patients with metabolic syndrome. Nevertheless, additional monitoring of hepatic alterations during lomitapide therapy is highly recommended.

Patients receiving lomitapide were on a low‐fat diet with <20% energy from fat, with vitamin E and essential fatty acids supplemented to maintain normal levels. Data from the phase 3 trial, were collected on the fat‐soluble vitamins A, D, E and K, beta‐carotene and essential fatty acids. During the study period, there were no deficiencies in any of these key micronutrients and some increased slightly, indicating that the supplementation levels were sufficient, and no additional fat‐soluble vitamins required supplementation. The observed decrease in the mean levels of vitamin E from baseline to weeks 26 and 78 is not unexpected given the profound lipid‐lowering effect with lomitapide and the fact that vitamin E is transported to the peripheral tissues via LDL‐C.^52^ Importantly, the ratio of vitamin E: total lipids did not decrease and none of the patients had a ratio <1.0 at any time during the study. Mean levels of beta‐carotene remained relatively stable and mean omega‐3 and ‐6 fatty acids were above the upper limit of normal at baseline, consistent with additional dietary supplementation. Levels of these fatty acids decreased to within or just above the normal range at weeks 26 and 78. Overall, no clinically meaningful changes were observed in fat‐soluble vitamins or essential fatty acids during lomitapide treatment.

At baseline, patients in the Italian cohort had high LDL‐C levels (mean [IQR] 276.5 mg/dl [196.0–386.2 mg/dl]), despite having access to statins, ezetimibe, apheresis and PCSK9 inhibitors. More than half of patients had experienced a major adverse cardiovascular event by the time of their pre‐lomitapide baseline assessment. This represents a population with a high risk of cardiovascular events driven by very high cholesterol levels that are not controlled with standard lipid‐lowering therapy, which is a classic hallmark of HoFH. We noted in this cohort that only 35% were receiving apheresis at baseline, which is lower than that documented in some past studies.^22^, ^53^, ^54^ This could be due to the fact that apheresis is not widely available, and many patients need to travel substantial distances for treatment. Furthermore, it is possible that not all patients agree to undergo this treatment due to the potential impact on their quality of life.^55^ Alongside the effective LDL‐C lowering, eight patients (67%) were able to stop LA after commencing lomitapide treatment.

The present study has several limitations. Validation of CK‐18, CK‐18 fragments and ELF have been conducted in NAFLD and NASH.^28^, ^29^, ^30^, ^56^ The very fact that biomarkers can differentiate between NAFLD and NASH^30^, ^31^, ^32^ indicates that the manifestations of changes in hepatic biomarkers can be defined by aetiology. Importantly, however, these biomarkers are not validated under the conditions of the current trial. Whereas these biomarkers have largely been evaluated in cross‐sectional comparisons involving patients with biopsy‐proven steatosis, the current study was a prospective interventional trial in which patients did not have pre‐existing steatosis at baseline and only a subset developed steatosis over the course of the study. Due to the retrospective nature of the study, particularly for the Italian cohort, laboratory protocols may differ between sites. Samples may be supplied as either serum or plasma, and individual local procedures may differ. Another limitation of the present analysis is the lack of even longer‐term follow‐up data in more patients. Although liver data were recorded for more than 9 years in some patients, the natural history of liver steatosis can be highly variable and takes time to manifest in patients, as only a subset of patients in the general population with NAFLD go on to develop steatohepatitis or fibrosis.^57^, ^58^ In addition, fibrosis can take upwards of 7 years to become evident, and end‐stage disease such as cirrhosis taking 15 or more years to develop.^59^ Indeed, among the 24 patients with calculable FIB‐4 scores, 10 had follow‐up of less than 24 months, which may be insufficient time to observe the development of fibrosis. Certainly, longer‐term follow‐up in a larger group of patients is warranted.

In conclusion, the MTP inhibitor lomitapide can cause temporary elevations in liver transaminases and mild to moderate accumulation of hepatic fat. Across the data sets analysed, biomarkers of hepatic inflammation or fibrosis do not suggest progressive liver disease over the course of the study and hepatic elastography remained normal after more than 9 years of lomitapide treatment. HoFH patients are at high risk of CVD events and premature mortality due to their extremely high LDL‐C exposure since birth.^60^ Therefore, the benefit/risk ratio of treating HoFH patients with lomitapide remains strongly in favour of treatment. In addition, the reduction or avoidance of LA treatment is an additional benefit. Lomitapide can be considered an effective LDL‐C‐lowering therapy for the long‐term treatment of HoFH with an acceptable liver safety profile. Longer‐term follow‐up of these patients will continue to provide valuable effectiveness and safety information.

## FUNDING INFORMATION

The phase 3 trial, extension phase study and the LOWER registry were funded by Amryt Pharmaceuticals DAC. The Italian study was funded as an investigator‐initiated study grant from Amryt Pharma.

## CONFLICT OF INTEREST

DL has received consultancy fees and/or honoraria from Amryt, Sanofi and Aegerion. LD has received consultancy fees and/or honoraria from Amgen, Akcea, Amryt, Pfizer, SOBI, Aurora Biopharma and Sanofi. SOB is an employee of Amryt Pharmaceuticals DAC. MA has received consultancy fees and/or honoraria from Akcea/Ionis, Alfasigma, Amgen, Amryt, Daichi‐Sankyo, Novartis, Pfizer, Regeneron, Sanofi.

## ETHICS APPROVAL

The studies described were performed in accordance with the Helsinki Declaration of 1964, and its later amendments. All studies received institutional review board approval. All patients provided informed consent for study participation and publication of related data.

## REGISTRATION

Phase 3 trial: NCT00730236 reg. 8 August 2008; extension: NCT00943306 reg. 22 July 2009; LOWER: NCT02135705 reg. 12 May 2014.

## Supporting information


Data S1
