# Supplementary material for: Long‐term hepatic safety of lomitapide in homozygous familial hypercholesterolaemia
Source: Liver Int. 2022 Dec 30;43(2):413–23. doi: 10.1111/liv.15497 (PMC10107656; doi:10.1111/liv.15497)
Supplement: Supplementary file 1 — Data S1 [file LIV-43-413-s001.docx]

**Supplementary Information**

**Title:** Long-term hepatic safety of lomitapide in homozygous familial hypercholesterolaemia

**Journal:** Liver International

**Authors:** Dominique Larrey,^1^ Laura D’Erasmo,^2^ Sallyann O’Brien,^3^ Marcello Arca^2^

and the Italian Working Group on Lomitapide

**Affiliations:**

1. University Hospital of Montpellier, 191 Avenue du Doyen Gaston Giraud,
   34295 Montpellier CEDEX 5, France
2. ‘Sapienza’ University of Rome, Viale dell’Università 37, Rome 00161, Italy
3. Amryt Pharmaceuticals DAC, 4 Mespil Road, Dublin 4, Ireland

**Author for correspondence:**

Dominique Larrey

Liver Unit

Saint-Eloi Hospital

80 Avenue Fliche

34295 Montpellier CEDEX 5

France

Tel. +33 04 67 33 70 81

Email: dom-larrey@chu-montpellier.fr

**Supplemental Table 1.** Datasets analysed

| **Cohort** | **Patients, n** | | | | | | |
| --- | --- | --- | --- | --- | --- | --- | --- |
|  | **Trans-aminases** | **Hy’s law** | **CK-18** | **ELF** | **FIB-4** | **Hepatic imaging** | **Fat-soluble vitamins and essential fatty acids** |
| Phase 3 trial (N=29) | 29 | 29 | 26 | 26 | – | – | 29 |
| LOWER (N=214) | 214 | 214 | – | – | – | – | – |
| Italian cohort (N=34) | 34 | 2 | – | – | 25 | 28 | – |

CK-18, cytokeratin-18 and CK-18 fragments; ELF, enhanced liver fibrosis score; LOWER, Lomitapide Observational Worldwide Evaluation Registry

**Supplemental Figure 1**. Mean body weight and BMI in patients receiving lomitapide in the Phase 3 study

**
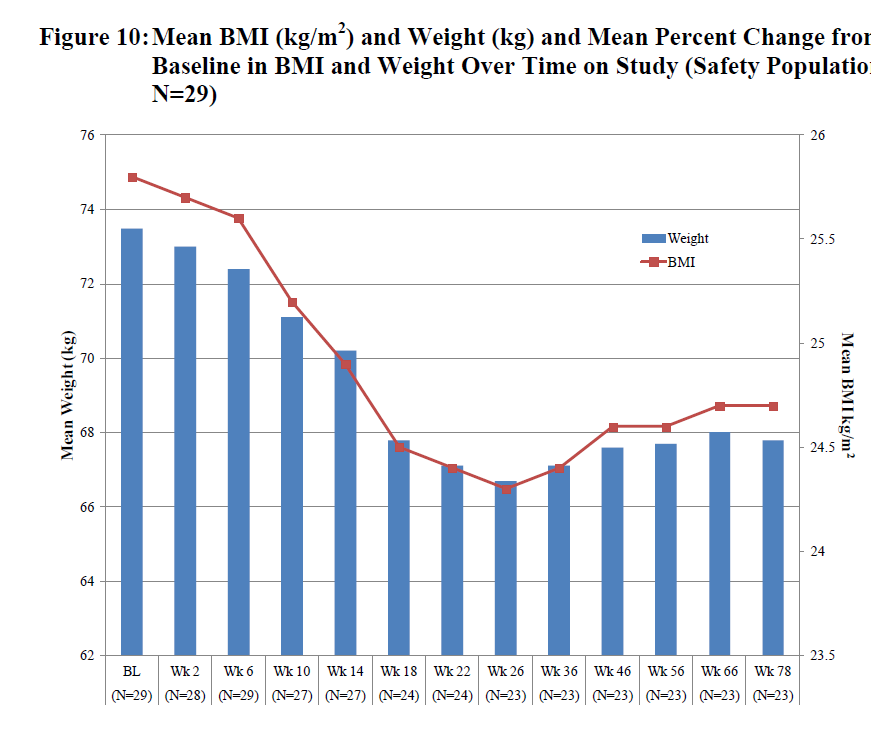
**

BL, baseline; BMI, body mass index; wk, week

**Supplemental Table 2.** Patient data on elevated liver function tests and Hy’s law cases at any time during the efficacy and safety phase from the Phase 3 clinical trial

| **Subject ID** | **ALT** | | **AST** | |
| --- | --- | --- | --- | --- |
|  | **Frequency ≥3xULN, <5xULN, n** | **>5xULN, n** | **Frequency ≥3xULN, <5xULN, n** | **Frequency >5xULN, n** |
| 01-001 | 0 | 0 | 0 | 0 |
| 01-002 | 0 | 0 | 0 | 0 |
| 01-003 | 0 | 0 | 0 | 0 |
| 01-004 | 6 | 5* | 4 | 0 |
| 01-006 | 0 | 0 | 0 | 0 |
| 02-001 | 1 | 0 | 0 | 0 |
| 02-002 | 1 | 3 | 1 | 0 |
| 11-001 | 0 | 0 | 0 | 0 |
| 11-002 | 0 | 0 | 0 | 0 |
| 11-003 | 0 | 0 | 0 | 0 |
| 11-004† | 5 | 0 | 0 | 0 |
| 12-001 | 2 | 0 | 1 | 0 |
| 12-003 | 0 | 0 | 0 | 0 |
| 12-004 | 1 | 1 | 1 | 0 |
| 12-005 | 0 | 0 | 0 | 0 |
| 12-006 | 0 | 0 | 0 | 0 |
| 13-001† | 0 | 0 | 0 | 0 |
| 13-002 | 0 | 0 | 0 | 0 |
| 22-003 | 0 | 0 | 0 | 0 |
| 22-004 | 2 | 0 | 1 | 0 |
| 23-001 | 1 | 0 | 0 | 0 |
| 23-002 | 0 | 0 | 0 | 0 |
| 23-003 | 0 | 0 | 0 | 0 |
| 31-001 | 1 | 0 | 0 | 0 |
| 31-002 | 0 | 0 | 0 | 0 |
| 32-001 | 0 | 1 | 0 | 1 |
| 32-002 | 0 | 0 | 0 | 0 |
| 33-001 | 0 | 0 | 0 | 0 |
| 35-001 | 0 | 0 | 0 | 0 |
| **Hy’s Law analysis (n=29)** | | | | **Patients, n (%)** |
| ALT or AST elevations ≥3xULN | | | | 10 (34.5) |
| Bilirubin elevations ≥2xULN | | | | 2 (6.9) |
| ALT or AST ≥3xULN and bilirubin ≥2xULN (meets Hy’s law) | | | | 0 (0) |

*One ALT reading during the efficacy and safety phase was >10xULN, <20xULN

†AST or ALT >3xULN during screening phase or at baseline (prior to receiving lomitapide) ALT, alanine aminotransferase; AST, aspartate aminotransferase; ULN, upper limit of normal

**Supplemental Figure 2.** Time to first elevated LFT (ALT/AST ≥3x ULN or bilirubin ≥2x ULN) from the 8-year LOWER registry

**
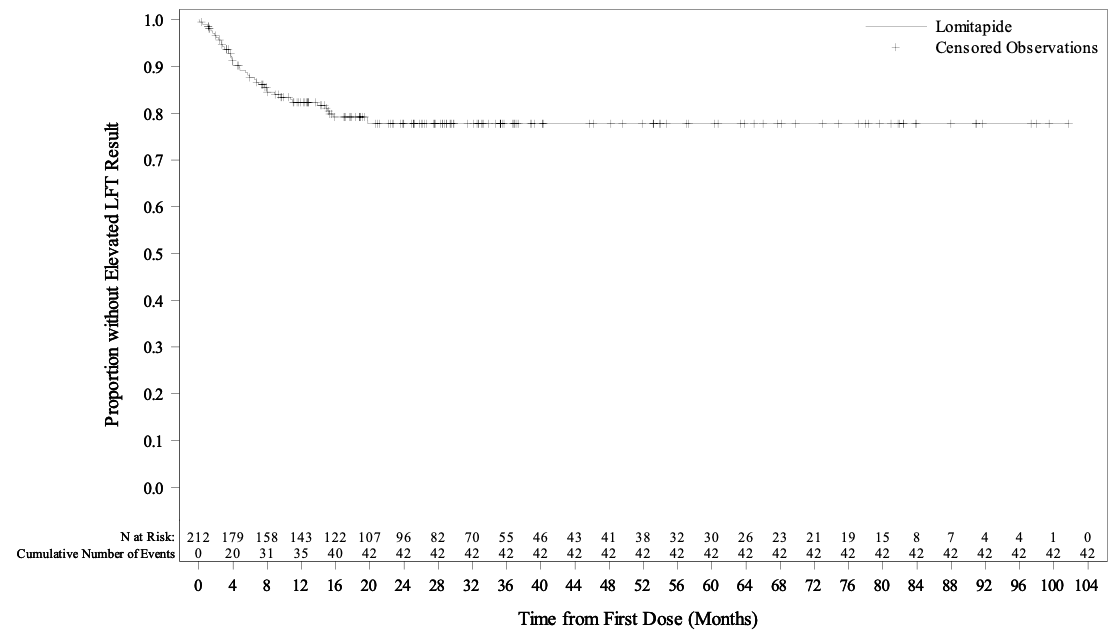
**

ALT, alanine aminotransferase; AST, aspartate aminotransferase; LFT, liver function test, ULN, upper limit of normal

**Supplemental Table 3**. Incidence of hepatic transaminase elevations and Hy’s law from the 8-year LOWER registry

| **Parameter** | **Unadjusted incidence,  n (%) [95%CI]** | **Exposure-adjusted incidence rate [95%CI]** |
| --- | --- | --- |
| Number of patients | N=214 | PY=602.72 |
| Elevation of hepatic transaminases >3x ULN that persist greater than 4 weeks despite dose reduction or interruption | 19 (8.9) [5.4-13.5] | 3.2 [1.9-4.9] |
| Hy’s law criteria | 0 [0.0-1.7] | 0.0 [0.0-0.6] |

CI, confidence interval; ESI, event of special interest; PY, person years; ULN, upper limit of normal

Data courtesy of the LOWER steering committee

**Supplemental Table 4.** Genetic mutation information by patient in the Italian cohort (n=34)

| **Subject ID** | **Mutation** | **Gene** | **Genotype** | **Functional status** |
| --- | --- | --- | --- | --- |
| 1 | not available | *LDLR* | HoFH | UNK |
| 2 | LDLR: exon 10: c.1567G>A, p.V523M (V502M) Pathogenic_Affects function, 12-25%LDLR activity in Hmz FH Kuwait, FH Bari-2 | *LDLR* | HoFH (homozygous) | DEF/DEF |
| 3 | LDLR: exon 14: c.2054C>T, p.P685L, (P664L)_Predicted to be Pathogenic, FH Gujerat, FH Frosinone1, FH Kanazawa-2, Disease Causing, FH Issoire / LDLR: exon 6: c.858C>A, p.S286 (S265R), Affects function FH Greece-2, Disease causing | *LDLR* | HoFH (double heterozygous) | DEF/DEF |
| 4 | not available | *LRLRAP1* | ARH | ARH |
| 5 | LDLRAP1: exon4: c.430_431insA, p.His144fs ARH 1, Affects Function | *LRLRAP1* | ARH | ARH |
| 6 | not available | *LDLR* | HoFH (homozygous) | UNK |
| 7 | LDLRAP1: exon4: c.430_431insA, p.His144fs ARH 1, Affects Function | *ARH* | ARH | ARH |
| 8 | LDLR: exon 7: c.974G>A, p. C325Y (C304Y) , Disease Causing / LDLR dup exon 7-15 | *LDLR* | HoFH (compound heterozygous) | UNCLASSIFIED |
| 9 | LDLR: exon 1: c.-156C>T , p.? Probably affects function | *LDLR* | HoFH (homozygous) | UNCLASSIFIED |
| 10 | LDLRAP1: exon4: c.430_431insA, p.His144fs ARH 1, Affects Function | *LRLRAP1* | ARH | ARH |
| 11 | LDLR: exon 12: c.1775G>A, p.G592E (G571E) Disease Causing_Affects Function_FH Sicily, Foggia-1, FH Naples-4_/ LDRL: exon14: c.2054C>T, p.P685L (P664L) Disease Causing FH Gujerat, FH Frosinone1, FH Kanazawa-2_ | *LDLR* | HoFH (compound heterozygous) | DEF/DEF |
| 12 | LDLR: exon 10: c.1567G>A, p.V523M (V502M) Pathogenic_Affects function, FH Kuwait, FH Bari-2 | *LDLR* | HoFH (homozygous) | DEF/DEF |
| 13 | LDLRAP1: exon4: c.430_431insA, p.His144fs ARH 1, Affects Function | *LDLRAP1* | ARH | ARH |
| 14 | LDLR: exon 3: c.304C>T, p.Gln102* (Q81X) FH Raponi, Affect Function / LDLR: exon 5: c.718G>A, p.E240K (E219K) , Disease Causing_Probably Affects Function_ FH Charlotte | *LDLR* | HoFH (compound heterozygous) | NULL/DEF |
| 15 | LDLRAP1: exon4: c.430_431insA, p.His144fs ARH 1, Affects Function | *LDLRAP1* | ARH | ARH |
| 16 | LDLR: exon 8: c.1135T>C, p.C379R (C358R), Disease Causing_Affects function_FH Naples-1 | *LDLR* | HoFH (homozygous) | DEF/DEF |
| 17 | LDLR: exon 11: c.1646G>A, p.G549D (G528D) Pathogenic _Affects function_FH Palermo-1 / LDLR: exon 12: c.1739C>T (p.S580F), Likely Pathogenic | *LDLR* | HoFH (compound heterozygous) | UNCLASSIFIED |
| 18 | LDLR: exon 11: c.1646G>A, p.G549D (G528D) Pathogenic FH Palermo-1_Affects function | *LDLR* | HoFH (homozygous) | NULL/NULL |
| 19 | LDLR: exon 9: c.1291G>A, p.A431T (A410T) Disease Causing_Affects Function_ FH Algeria-2 (22% LDLR on cell surface, 20% LDL binding and internalization in transfected COS cells) | *LDLR* | HoFH (homozygous) | DEF/DEF |
| 20 | LDLR new mutation of exon 4 deletion of exon 9 | *LDLR* | HoFH (compound heterozygous) | UNCLASSIFIED |
| 21 | LDLRAP1: 2i : c.89-1 G>C, p.K30Tfs*3 Pathogenic | *LDLRAP1* | ARH | ARH |
| 22 | LDLR: 15i: c.2311+1G>A, p.?_Affects Function _FH Benevento | *LDLR* | HoFH (homozygous) | DEF/DEF |
| 23 | LDLR: exon 8: c.1109A>C, p.N370T (N349T), Disease Causing_Probably affects function, FH Reggio Calabria | *LDLR* | HoFH (homozygous) | UNCLASSIFIED |
| 24 | LDLR: exon 11: c.1646G>A, p.G549D (G528D) Pathogenic FH Palermo-1_Affects Function / LDLR: Del e13-15, 5kb , Pathogenic_FH Palermo-3 | *LDLR* | HoFH (compound heterozygous) | NULL/NULL |
| 25 | LDLR: exon 12: c.1775G>A, p.G592E (G571E) Disease Causing_Affects Function_ FH Sicily, Foggia-1, FH Naples-4 (Functional studies demonstrate that this variant would lead to deficient LDLR protein (Susan-Resiga et al., 2017)) | *LDLR* | HoFH (homozygous) | DEF/DEF |
| 26 | LDLR: exon 4: c.373C>T, p.Q125X (Q104X) Affects Function FH Foggia-2 / PCSK9: exon 1: c.60_ 65dupGCTGCT Indel | *LDLR* | HoFH (double heterozygous) | UNCLASSIFIED |
| 27 | LDLR: exon 11: c.1618G>A , p.A540T (A519T) Disease Causing_Probably affects function/ LDLR: exon 12: c.1775G>A, p.G592E (G571E) Disease Causing_Affects function_FH Sicily, Foggia-1, FH Naples-4 | *LDLR* | HoFH (compound heterozygous) | UNCLASSIFIED |
| 28 | LDLR: exon 12: c.1775G>A, p.G592E (G571E) Disease Causing_Affects Function_ FH Sicily, Foggia-1, FH Naples-4 (Functional studies demonstrate that this variant would lead to deficient LDLR protein (Susan-Resiga et al., 2017)) | *LDLR* | HoFH (homozygous) | DEF/DEF |
| 29 | LDLR: exon 8: c.1135T>C, p.C379R (C358R), Disease Causing_Probably Affects Function_ FH Naples-1 / LDLR: exon 9: c.1195C>A, p.A399T (A378T), Disease Causing_Probably Affects Function, FH Nuoro | *LDLR* | HoFH (compound heterozygous) | UNCLASSIFIED |
| 30 | LDLR exon 3: c.265T>C , p.C89R (C68R) , Probably Affect Function / LDLR: exon 12: c.1775G>A, p.G592E (G571E) Disease Causing_Affects function_FH Sicily | *LDLR* | HoFH (compound heterozygous) | DEF/DEF |
| 31 | LDLR: exon 6: c.828C>G, p.C276W (C255W) Pathogenic_Probably affects function, FH Sassari-3 | *LDLR* | HoFH (homozygous) | UNCLASSIFIED |
| 32 | LDLR: 67+1_68-1_(1845+1_1846-1) Del, Null protein | *LDLR* | HoFH(homozygous) | NULL/NULL |
| 33 | LDLR: ex2-12del FH Pavia | *LDLR* | HoFH (homozygous) | NULL/NULL |
| 34 | LDLR: exon 4: c.641 G>A / LDLR: exon -1: c.-188C>T, p.? | *LDLR* | HoFH (compound heterozygous) | UNCLASSIFIED |

HoFH, homozygous familial hypercholesterolaemia; UNK, unknown

**Supplemental Figure 3.** ELF score during lomitapide treatment in the long-term extension study

**
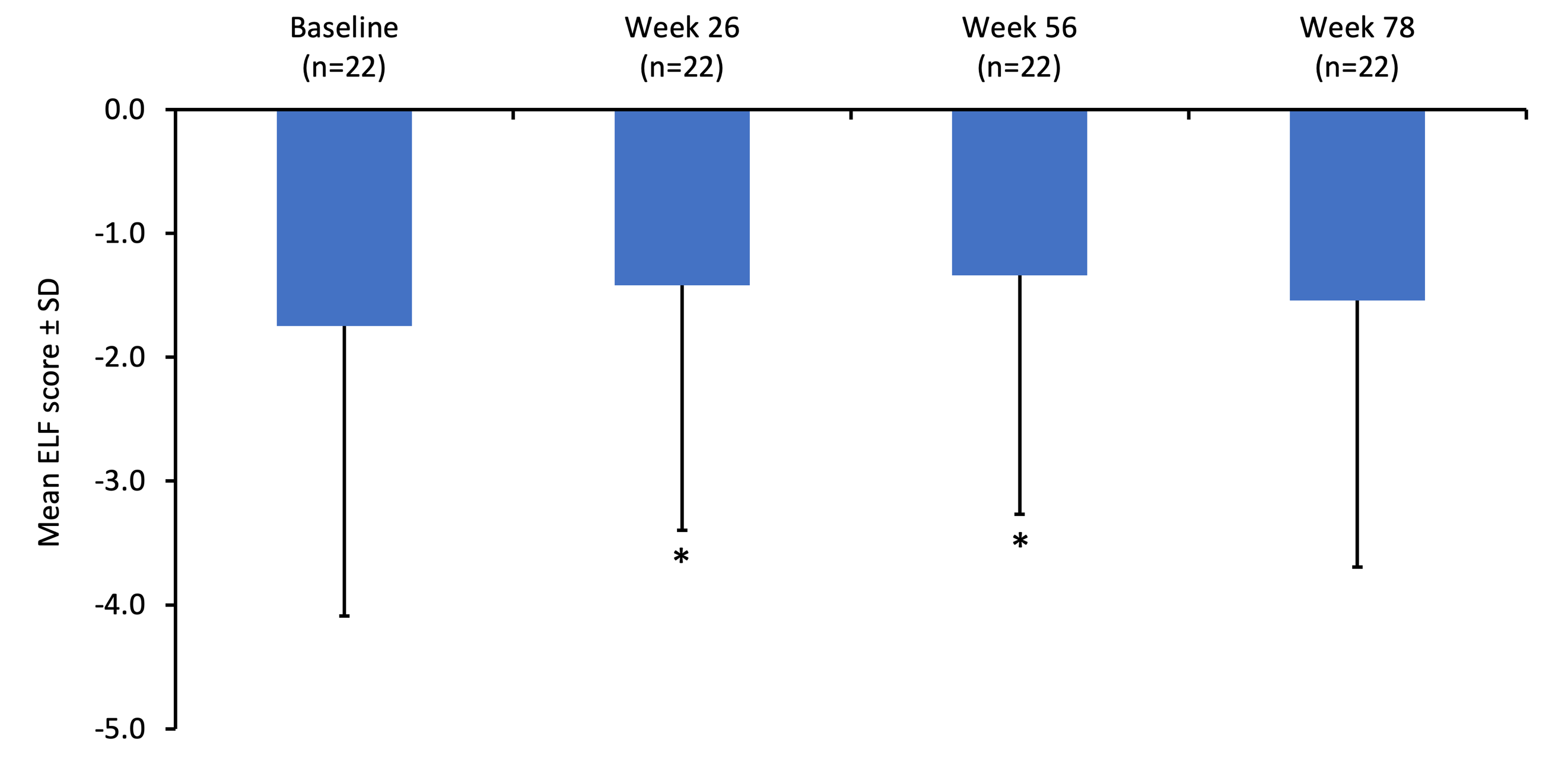
**

Values represent mean ± SD in patients with follow-up data

*p<0.05

ELF, enhanced liver fibrosis; SD, standard deviation

**Supplemental Table 5.** Baseline and follow-up hepatic steatosis for patients with baseline and/or follow-up ultrasound data in the Italian cohort (n=28)

| **Individual patient data by months of exposure to lomitapide** | | | | | |
| --- | --- | --- | --- | --- | --- |
| **Subject ID** | **Lomitapide exposure, months** | **Lomitapide dose at follow-up, mg/day** | **Baseline US result** | **Follow-up US result** | **Degree of change** |
| 20 | 1.4 | 10 | absent | absent | - |
| 34 | 4.0 | 10 | - | mild | N/A |
| 14 | 4.7 | 5 | mild | mild | - |
| 3 | 11.2 | 10 | mild | mild | - |
| 2 | 11.5 | 5 | mild | moderate | ↑ |
| 11 | 12.3 | 10 | absent | absent | - |
| 4 | 14.1 | 5 | absent | mild | ↑ |
| 19 | 15.3 | 10 | - | absent | N/A |
| 10 | 17.9 | 5 | - | moderate | N/A |
| 31 | 18.4 | 20 | absent | mild | ↑ |
| 12 | 19.4 | 20 | moderate | moderate | - |
| 8 | 24.8 | 5 | - | moderate | N/A |
| 17 | 25.7 | 40 | mild | mild | - |
| 16 | 31.3 | 20 | mild | mild | - |
| 7 | 32.0 | 5 | - | moderate | N/A |
| 33 | 33.5 | 20 | absent | absent | - |
| 26 | 33.9 | 30 | mild | mild | - |
| 6 | 35.8 | 20 | absent | absent | - |
| 9 | 36.3 | 5 | absent | absent | - |
| 15 | 40.4 | 40 | moderate | moderate | - |
| 13 | 41.3 | 10 | absent | moderate | ↑↑ |
| 5 | 41.7 | 5 | - | moderate | N/A |
| 32 | 52.3 | 20 | absent | absent | - |
| 18 | 55.1 | 40 | absent | moderate | ↑↑ |
| 22 | 56.7 | 30 | absent | absent | - |
| 24 | 109.9 | 20 | absent | moderate | ↑↑ |
| 27 | 110.4 | 40 | absent | moderate | ↑↑ |
| 29 | 117.4 | 60 | mild | mild | - |
| **Analysis of all patients with ultrasound data at baseline and follow-up (n=22)** | | | | | |
| **Patient group** | |  | **Patients, n (%)** |  |  |
| All patients | |  | 22 (100) |  |  |
| No change | |  | 15 (68.2) |  |  |
| Increase 1 degree | |  | 3 (13.6) |  |  |
| Increase 2 degrees | |  | 4 (18.2) |  |  |
| Decrease 1 degree | |  | 0 (0.0) |  |  |
| Decrease 2 degrees | |  | 0 (0.0) |  |  |
| All increases | |  | 7 (31.8) |  |  |
| All decreases | |  | 0 (0.0) |  |  |

Degree of steatosis is given according to the criteria of local investigators; N/A indicates patients with missing data at baseline. Number of arrows denote degree of change.

N/A, not applicable; US, ultrasound

**Supplemental Figure 4**. Graphical representation of baseline and follow-up hepatic steatosis for patients with baseline and follow-up ultrasound data in the Italian cohort (n=22)


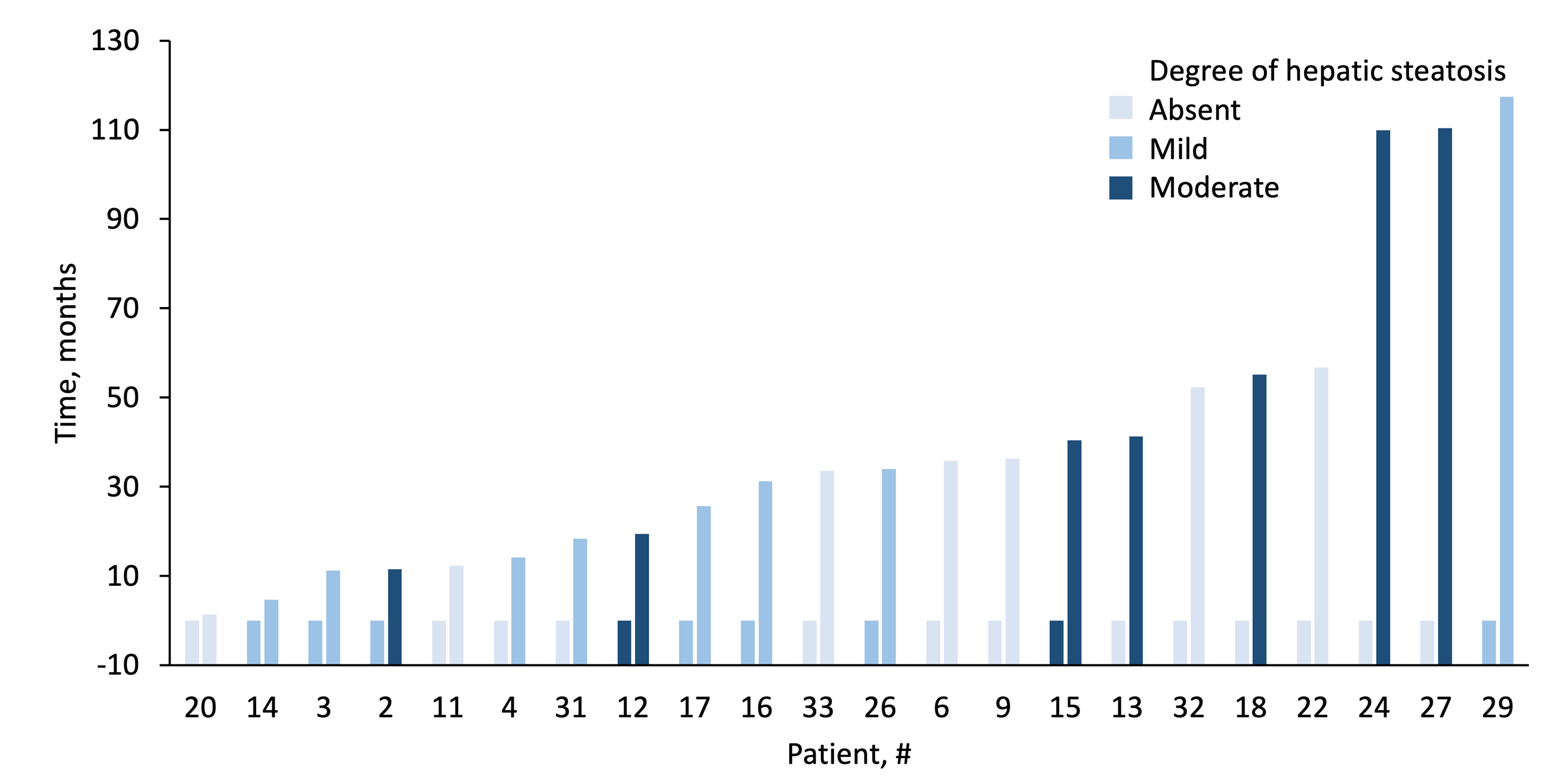


Degree of steatosis is given according to the criteria of local investigators; bars represent individual patient data at baseline and follow-up

**Supplemental Table 6.** Hepatic stiffness and FIB-4 scores aligned by patient in the Italian cohort for patients with both FIB-4 and elastography data at follow up (n=16)

| **Subject ID** | **Hepatic stiffness carried forward, kPA** | **FIB-4 at follow-up** |
| --- | --- | --- |
| 7 | 3.5 | 2.79 |
| 4 | 3.6 | 2.32 |
| 26 | 4.0 | 0.82 |
| 12 | 4.3 | 1.23 |
| 5 | 4.5 | 1.40 |
| 16 | 4.6 | 4.65 |
| 13 | 4.8 | 0.62 |
| 15 | 4.9 | 0.87 |
| 14 | 5.1* | 1.03 |
| 31 | 5.1 | 1.21 |
| 33 | 5.2 | 0.43 |
| 8 | 5.4 | 1.38 |
| 32 | 5.5 | 0.70 |
| 24 | 5.9 | 0.38 |
| 27 | 6.1 | 0.38 |
| 23 | 6.2 | 0.70 |

*Patient with FIB-4 scores at follow up and no hepatic elastography at follow up have baseline elastography results reported

**Supplemental Table 7.** Observed values and changes from baseline to Week 26/LOCF and Week 78 for fat-soluble vitamins (Phase 3 study safety population)

| Variable [normal range] Time point | N | Baseline,  mean (SD) | Follow-up, mean (SD) | Change,  mean (SD) |
| --- | --- | --- | --- | --- |
| Vitamin A (µg/dL) [M: 38–93; F: 32–80 µg/dL] | | | | |
| Efficacy phase, Week 26/LOCF | 29 | 44.8 (16.91) | 50.0 (18.88) | 5.2 (10.37) |
| Safety phase, Week 78/LOCF | 23 | 48.5 (16.06) | 53.5 (13.52) | 5.0 (12.65) |
| Vitamin E (mg/dL) [M: 0.5–1.62; F: 0.5–1.73 mg/dL] | | | | |
| Efficacy phase, Week 26/LOCF | 29 | 2.9 (1.14) | 1.7 (0.64) | –1.2 (1.10) |
| Safety phase, Week 78/LOCF | 23 | 2.9 (1.19) | 1.7 (0.78) | –1.2 (1.31) |
| Vitamin E/total lipids ratio |  |  |  |  |
| Efficacy phase, Week 26/LOCF | 29 | 5.9 (2.42) | 6.0 (2.11) | 0.5 (2.18) |
| Safety phase, Week 78 | 23 | 5.5 (2.33) | 5.2 (1.42) | –0.3 (1.95) |
| 25 OH Vitamin D (ng/mL) [7–54 ng/dL] | | | | |
| Efficacy phase, Week 26/LOCF | 29 | 17.5 (11.18) | 24.5 (12.60) | 7.0 (9.47) |
| Safety phase, Week 78/LOCF | 23 | 18.4 (11.99) | 31.6 (18.11) | 13.2 (16.72) |
| Beta-carotene (µg/mL) [M: 0.04–0.59; F: 0.06–0.75 µg/mL] | | | | |
| Efficacy phase, Week 26/LOCF | 29 | 0.3 (0.24) | 0.2 (0.25) | –0.1 (0.19) |
| Safety phase, Week 78/LOCF | 23 | 0.3 (0.25) | 0.3 (0.35) | 0.0 (0.29) |
| Osteocalcin: uncarboxylated/total ratio*^¶^ | | | | |
| Efficacy phase, Week 26/LOCF | 29 | 24.9 (9.15)† | 25.2 (10.3) | 0.5 (8.17) |
| Safety phase, Week 78 | 23 | 24.9 (9.15)† | 29.3 (10.41) | 4.2 (10.85) |

*Normal range varies by gender and age; †baseline (Week 0) for n=28; ^¶^levels of vitamin K were assessed directly by measuring the ratio of uncarboxylated osteocalcin/total osteocalcin.

F; normal range for females; M, normal range for males; LOCF, last observation carried forward; SD standard deviation

**Supplemental Table 8.** Observed values and changes from baseline to Week 26 and Week 78 for fatty acids (Phase 3 study safety population)

| Variable [normal range] Time point | N* | Baseline,  mean (SD) | Follow-up, mean (SD) | Change,  mean (SD) |
| --- | --- | --- | --- | --- |
| Linoleic acid (µmol/L) [2270­–3850 µmol/L] | | | | |
| Efficacy phase, Week 26/LOCF | 28 | 4023.6 (1059.15) | 3289.9 (1056.85) | –745.5 (1116.35) |
| Safety phase, Week 78/LOCF | 22 | 4125.6 (1087.22) | 3764.8 (1326.56) | –405.5 (1323.70) |
| Alpha linolenic acid (µmol/L) [50–130 µmol/L] | | | | |
| Efficacy phase, Week 26/LOCF | 28 | 74.2 (50.95) | 47.2 (40.53) | -27.6 (46.21) |
| Safety phase, Week 78/LOCF | 22 | 76.8 (55.00) | 64.7 (61.53) | -17.8 (65.44) |
| EPA (µmol/L) [14–100 µmol/L] | | | | |
| Efficacy phase, Week 26/LOCF | 28 | 209.8 (127.96) | 82.4 (50.08) | –127.6 (127.80) |
| Safety phase, Week 78/LOCF | 22 | 222.7 (139.68) | 107.3 (95.16) | –119.2 (126.58) |
| DHA (µmol/L) [30–­250 µmol/L] | | | | |
| Efficacy phase, Week 26/LOCF | 28 | 348.2 (115.78) | 208.6 (83.24) | –138.6 (152.82) |
| Safety phase, Week 78/LOCF | 22 | 380.0 (157.44) | 251.8 (178.59) | –132.0 (173.59) |
| Arachidonic acid (µmol/L) [520–­1490 µmol/L] | | | | |
| Efficacy phase, Week 26/LOCF | 28 | 2148.1 (502.88) | 1205.9 (446.42) | –949.7 (696.85) |
| Safety phase, Week 78/LOCF | 22 | 2228.5 (505.47) | 1278.3 (673.75) | –959.3 (791.77) |
| Eicosatrienoic acid (µmol/L) [7–­30 µmol/L] | | | | |
| Efficacy phase, Week 26/LOCF | 28 | 20.9 (8.01) | 14.0 (7.73) | –6.8 (9.54) |
| Safety phase, Week 78/LOCF | 22 | 20.8 (8.08) | 12.3 (5.56) | –8.4 (10.39) |

*One patient was missing baseline values for these parameters

EPA, eicosapentaenoic acid; DHA, docosahexaenoic acid; LOCF, last observation carried forward; SD standard deviation
